# 

**Published:** 1880-01

**Authors:** 


### CONTENTS:

ARTICLE I.—Dental Fallacies. By John J. R. Patrick, D. D. S. ..... 385
ARTICLE II.—Justice—Injustice; the Ether Controversy. By Prof. Hodgkin ..... 398
ARTICLE III.—The Portrait of Crawford W. Long ..... 400
ARTICLE IV.—Proceedings of the Maryland and District of Columbia Dental Society. (Continued.) ..... 404
ARTICLE V.—Preparation of the Mouth for Artificial Dentures. By John Lauder, D. D. S. ..... 414
ARTICLE VI.—Heating Metals in Vacuo by the Electric Current. By T. A. Edison 418
ARTICLE VII.—Pittsburg Dental Association ..... 423

### EDITORIAL, ETC.

The New Departure Again—Letter from Prof. Henry S. Chase ..... 424
Dr. J. N. Farrar's Lectures ..... 425
Dies and Taps for Amateurs ..... 426

### BIBLIOGRAPHICAL.

The Importance of Preserving the Teeth and Dental Ethnography ..... 426
The Mouth and the Teeth. American Health Primer ..... 427
Physician's Visiting List for 1880. Lindsay & Blackiston ..... 427
Vick's Floral Guide for 1880 ..... 427
Atlas of Human Anatomy ..... 428
The Throat and Voice. American Health Primer ..... 428
The Scientific American ..... 428
Bartholow's Materia Medica and Therapeutics ..... 428
Illustrated Dictionary of Scientific Terms ..... 429

### OBITUARY.

Dr. Samuel Stockton White ..... 429

